# DISCRIMINATION AND ACCESS TO AGING AND LONG-TERM CARE SERVICES AND SUPPORTS FOR LGBTQIA+ OLDER ADULTS

**DOI:** 10.1093/geroni/igae098.1612

**Published:** 2024-12-31

**Authors:** Jason Flatt, Brittany Klenczar, Jalal Uddin, Max OHala, Erin Rook, Elise Hernandez, Karalin Sprague

**Affiliations:** University of Nevada Las Vegas, Las Vegas, Nevada, United States; University of Nevada Las Vegas, Las Vegas, Nevada, United States; University of Nevada Las Vegas, Las Vegas, Nevada, United States; University of Nevada Las Vegas, Las Vegas, Nevada, United States; University of Nevada Las Vegas, Las Vegas, Nevada, United States; SAGE, New York, New York, United States; SAGE, New York, New York, United States

## Abstract

By 2030, there will be over seven million LGBTQ+ adults in the U.S. who are aged 50+. Past research finds that LGBTQ+ older adults are less likely to reach out to providers and use aging-related and long-term care services because of fear of discrimination. Partnering with SAGE, a national advocacy and services organization for LGBTQ+ older adults, we conducted a national study, State of LGBTQ+ Aging Study, with LGBTQ+ older adults (n=643) across the U.S. We used descriptive statistics and bivariate analyses to examine associations between experiencing lifetime and everyday discrimination and concerns with access to aging-related and long-term care services. In terms of discrimination/victimization, over 41% (n=261) reported experiencing one or more lifetime discrimination events, 42% (n=263) reported experiencing violence due to being LGBTQ+, and 45% reported everyday discrimination. For aging-related and long-term care services, 28% reported planning for long-term care (e.g., family caregiving, nursing facilities, and home healthcare), 65% were concerned about accessibility of aging services, 64% were concerned about access to future medical care, 71% were concerned about access to future caregiving, and 76% were concerned about finding inclusive long-term care/caregiving resources. LGBTQ+ older adults who reported past LGBTQ+ violence, lifetime and everyday discrimination were more likely to report being concerned about accessibility of aging services, access to future medical care, and having a future caregiver (p-values <0.01). These findings highlight how discrimination may impact access to aging-related and long-term care services for LGBTQ+ older adults, and the need for future research and inclusive care efforts.

